# Behavior Analyst & Trainee Workloads: Baseline Reports, Ethical Implications, and Practical Solutions

**DOI:** 10.1007/s40617-025-01113-5

**Published:** 2025-12-10

**Authors:** Kimberly A. Schreck, Cameryn Padron, Timothy D. Caldwell, Samara J. Wilson

**Affiliations:** 1 Penn State Harrisburg, Behavioral Sciences & Education, 777 W Harrisburg Pike, W311 Olmsted Building, Middletown, PA 17057-4898 USA; 2 Vista Autism Services, Hershey, PA USA

**Keywords:** Workload, Behavior analysts, Behavior analyst trainees

## Abstract

Workload (e.g., direct client responsibilities, supervision, administration, and travel) within applied behavior analysis-based service provision may pose significant challenges for behavior analysts and trainees. Intensive workloads may result in overwork and related ethical issues, such as service and supervision quality issues, personal health issues, and availability of behavior analysts (e.g., absenteeism, turnover, and attrition). This survey of behavior analysts and trainees (*N* = 322) indicated that the average workload exceeded average employee expected workweek hours and recommended client assignments. Behavior analysts’ and trainees’ job responsibilities included many responsibilities beyond client services (e.g., supervision, administration, and travel). Many of these activities remained uncompensated. Respondents reported high levels of work and personal life stressors related to their workload. Owing to the multitude of possible ethical issues related to these workload factors, individuals, organizations, and the field of applied behavior analysis must continue to evaluate these workload variables and enact prevention, assessment, and intervention steps to mitigate overwork for the benefit of the profession and its clients.

**Supplementary Information:**

The online version contains supplementary material available at https://doi.org/10.1007/s40617-025-01113-5.

## Utility for Clinicians & Researchers

This study provides:

1. Baseline exploratory, descriptive reports of client assignments, workloads, and work and personal life stressors related to workload for behavior analysts and trainees.
2. Ethical implications of excessive workload.
3. Research-based recommendations for prevention, assessment, and intervention for work and personal life stressors for the individual, organizational, and field levels.

Across the United States, the demand and need for behavior analysts far outweighs the availability of professionals and may result in significant pressure to accept unmanageable workloads to fill this need (Behavior Analyst Certification Board [BACB], 2024b; Council of Autism Service Providers [CASP], 2020; Syed, 2024; Yingling et al., 2022). Extrapolation of data and past reports of behavior analysts’ client assignment numbers estimate averages of 25–100+ clients per behavior analyst (Syed, 2024; Yingling et al., 2022). These numbers far exceed past recommended client assignments of 6–15 clients per individual behavior analyst without an assigned Board Certified Assistant Behavior Analyst^®^ (BCaBA^®^) dependent upon the comprehensiveness of the treatment required and a typical 40-hour work week (CASP, 2020; Syed, 2024; Yingling et al., 2022).

More recently, recommendations for behavior analysts’ client assignments were changed to represent hours spent on direct (i.e., in presence of client) and indirect (i.e., not in presence of a client) tasks (CASP, 2024). The recommendations for completing these tasks were conceptualized within a multi-tiered responsibility model, with behavior analysts responsible for direct oversight of treatment implementation provided by others implementing direct intervention procedures. This recommendation model indicated that a behavior analyst working 40 hours per week may be able to provide approximately 35 hours per week (e.g., 150 recommended hours/4.3 weeks per month = 35 hours/week) of direct oversight of approximately 116–348 hours per week of treatment provided by others. Using estimates from this document of recommended individual client behavior analysis services of 10–40 hours per week (average 25 hours), this would equate to having direct oversight for approximately 5–14 clients per week which remains similar to previous recommendations (CASP, 2020; Syed, 2024; Yingling et al., 2022). Although these recommendations for time allocation suggest employers should consider travel, direct or indirect supervision, treatment comprehensiveness, and client progress within interventions, the recommendations did not provide direction or specific hour adjustments or client assignments related to comprehensiveness of treatment, behavior analysts’ travel, direct client contact, direct/indirect supervision, assessment, or treatment development and ongoing analysis.

Past recommendations for determining comprehensiveness of treatment while assessing the assigned number of clients or working hours for tasks varied on the basis of behavior analysts’ responsibilities for each case (CASP, 2020; Syed, 2024; Yingling et al., 2022). Behavior analysts assigned clients who had more complex behaviors requiring more treatment hours per week (e.g., aggression, self-injury, etc.), who required direct supervision of treatment and staff, who required travel (e.g., home and community services), who had needs or expertise at the cusp of the behavior analyst’s competency, who lived in complex social situations, or who required additional administrative support should receive fewer client assignments (CASP, 2020, 2024). Additionally, these past recommendations indicated that for every 10 hours of direct treatment implementation, behavior analysts should spend 1–2 hours on development and continual assessment of interventions (CASP, 2020, 2021; Syed, 2024). These client complexity, additional responsibilities, and time commitment considerations should influence calculations for deciding the assigned number of clients for a behavior analyst. Although LeBlanc and colleagues (LeBlanc et al., 2019) have developed a method for assessing individual behavior analysts’ time management, travel requirements, and match of clinical skills to client needs, no industry recommendations were found to indicate how decisions should be made to assign behavior analysis trainees’ client assignments or workloads, other than the BACB fieldwork hour maximum requirements of 130 hours per month (BACB, 2019).

In addition to client complexity and responsibilities influencing numbers of assigned clients, administrators and employers must consider behavior analysts’ daily work responsibilities when assigning clients. Although brief behavior analyst job descriptions exist related to providing treatment (i.e., implementing ABA-based intervention), supervision, and fieldwork requirements (BACB, 2019, 2024a), behavior analysts’ and trainees’ other responsibilities remain relatively undocumented. Behavior analysts and trainees may complete a variety of other responsibilities, including (a) administration (e.g., auditing, bookkeeping, billing, report writing, insurance authorizations), (b) direct client care (e.g., assessment, treatment implementation), (c) treatment plan development and analysis, (d) collaboration (e.g., navigating complex care coordination), and (e) training/supervision (CASP, 2024; Layden et al., 2024). Balancing client-specific and general work responsibilities should influence workload (e.g., hours, number of clients) and compensation for workload. At this time, no research exists to determine the baseline or decision making for how or if these and other related variables (e.g., types of tasks, time required for tasks, complexity of client issues) influence behavior analysts’ and trainees assigned workload.

If behavior analysts or trainees receive unmanageable assigned workloads, several ethical implications may occur. A combination of complex clients, excessive daily work responsibilities, and a high number of client assignments during work hours may impact both job performance and personal lives. Workloads may impact service and supervision quality (e.g., inaccurate or missed data collection and analysis, neglecting necessary program changes, decreased productivity, lack of support: Jimenez-Gomez et al., 2021; Novack & Dixon, 2019; Plantiveau et al., 2018; Syed, 2024); personal health and well-being (e.g., exhaustion, decreased problem solving capacity, lack of empathy, depression: Bottini et al., 2020; Brown, 2021; Dounavi et al., 2019; Jimenez-Gomez et al., 2021; Kranak, 2022; Novack & Dixon, 2019; Plantiveau et al., 2018; Slowiak & DeLongchamp, 2022); and availability of behavior analysts and trainees (e.g., low job satisfaction, absenteeism, turnover, and attrition: Dounavi et al., 2019; Kazemi et al., 2022; Kranak, 2022; Novack & Dixon, 2019; Plantiveau et al., 2018; Slowiak & DeLongchamp, 2022).This research indicating the impacts of excessive workload suggested that overworked behavior analysts and trainees may engage in behavior leading to ethical issues according to the Ethics Code for Behavior Analysts (BACB, 2020). These issues may relate to accountability for the number of clients they can effectively accept (Section 1 Responsibility as a Professional and Section 3 Responsibility to Clients and Stakeholders), to the timeliness, effectiveness, and continuity of their treatments (Section 2 Responsibility in Practice) and to their supervisory volume and accountability (Section 4 Responsibility to Supervisees and Trainees).

Although past literature indicated that behavior analysts’ workloads impacted their work performance, personal lives, and possibly ethical implementation of services, significant questions remain related to the baselines (i.e., current reported workloads and time spent on tasks), interactions, and impacts of behavior analysts’ and trainees’ current tasks, assigned client numbers, workloads, and reinforcement (i.e., compensation). In this manuscript, we report descriptive, exploratory data for behavior analyst and trainee respondents’ baselines for reported current workloads (e.g., hours, numbers of clients, and types of tasks), compensation for work completed (e.g., payment, billable activities), relationships among workload and client complexity (e.g., behaviors, goals, interventions), and reported personal issues related to workload. In addition to determining baselines and relationships among workload variables, we provide suggestions for prevention, assessment, and intervention.

## Method

### Respondents

Each potential respondent received an email, social media posting, or direct message to participate in an online survey. Those who responded were included if they indicated they were a practicing behavior analyst or a trainee. Each respondent could select multiple practicing credentials. Thus, the total for all credentials (i.e., *N* = 384) does not equal the total number of individual respondents (i.e., *N* = 322). Individuals practicing behavior analysis (*N* = 322, BCBA^®^ *n* = 220, 67.1%; BCBA-D^®^ *n* = 25, 7.6%; licensed psychologist *n* = 1 , 0.3%; trainees *n* = 65, 9.8%; state-licensed behavior analyst or equivalent *n* = 73, 22.3%) were condensed into two variables (i.e., behavior analyst or trainee) for analysis. To allow for further analysis of results, respondents who indicated practicing behavior analysis at any time were categorized into a group as a practicing behavior analyst (i.e., BCBA^®^, BCBA-D^®^, licensed psychologist, state-licensed behavior analyst) or as a trainee (i.e., student completing applied behavior analysis [ABA] fieldwork).

### Materials/Measures

Literature reviews and personal experience were used to self-develop questions of interest in each questionnaire domain (see Appendix for questionnaire). Questions were reviewed for face and content validity by two doctoral level behavior analysts with a combined thirty plus years of experience in the field and one trainee. Content validity included review of empirical research, business practices, clinical experience, and professional requirements; for example, literature was reviewed related to work and personal life stressors, organizational requirements for work tasks, and discipline descriptions of work descriptions (e.g., BACB, 2024a, b; Layden et al., 2024; CASP, 2024). The questionnaire was divided into separate sections: (1) Demographic information (e.g., credentials, years of experience, age, gender, practicing state, job environment), (2) Workload (e.g., hours worked, number of clients, types of tasks), (3) Compensation for tasks (e.g., payment, billable activities), (4) Client complexity (e.g., client behaviors, goals, interventions) and (5) Reported issues related to workload (e.g., work and personal stressors). The overall number of questions for all sections totaled 28.

The questionnaire asked respondents questions about demographic information, including personal information, credentialing, and clinical practice. Personal information included age and gender identity (e.g., male, female, nonbinary). Credentialing questions included clinical credentials (e.g., BCBA^®^, Licensed Psychologist, trainee), time since certification, or credentials to practice (e.g., range from not yet certified to 10+ years post certification). Clinical practice information included if they worked in clinical practice in the last five years, where they worked (e.g., states), and what type of environment they worked in (e.g., private school, consultation, private ABA consulting, and residential treatment facilities).

To determine workload, respondents were asked about the average hours they worked per week, the number of clients assigned to them in the last year for direct and for indirect oversight, their highest number of assigned clients, and the hours they engaged in specific tasks per week. The average number of hours respondents required to complete non-face-to-face tasks (e.g., analyzing graphs, developing behavior plans, direct services, administration, collaboration) was recorded according to the average number of hours completed for each task per week. For the hours completed per week, respondents provided how they received their pay (e.g., salary, hourly), if they received overtime pay, and how or if specific tasks were compensated.

In addition to types of tasks, respondents were asked questions related to the complexity of their clients. These questions included types of severe problem or challenging behavior (e.g., aggression, self-injurious behavior), non-severe behavior (e.g., elopement, non-significant property destruction, screaming), skill acquisition (e.g., skill acquisition goals, verbal ability, advanced communication, or problem-solving goals), other co-occurring issues (e.g., medical fragility, specialty skill instruction such as feeding, toileting), and classroom-wide interventions. Respondents provided an average number of how many clients they served with each behavior, skill, or co-occurring issue on a sliding scale ranging from 0 to 250 clients.

After completing workload related information, respondents indicated the existence of work and personal life stressors. These questions indicated the presence of multiple work and personal life issues, such as consideration of changing jobs or fields, reduced job satisfaction, personal ethical dilemma(s), and personal distress.

### Procedure

The questionnaire was uploaded to Qualtrics. Institutional review board approval was obtained from the Penn State Office for Research Protections. Upon receiving designation of an exempt protocol, respondents were recruited via emails (e.g., listserv, Association of Professional Behavior Analysts, Verified Course Sequence Instructors/Universities, personal members of organizations) and social media sites with the permission of the page owner or group administrator (e.g., LinkedIn, Facebook, Instagram). Participants received an email recruitment letter or saw the recruitment post on social media stating the importance of the study, questionnaire procedures, and a link for survey completion. Respondents were asked to forward emails and ads to their companies/colleagues. Owing to the forwarding of the emails and ads, it was not possible to determine the percentage of those who received the information about the survey compared to those who completed it. Completion of the study took an estimated ten minutes to complete with respondents receiving no compensation for completing the questionnaire.

### Data Analysis

#### Participant Demographics

Descriptions of the respondents related to a variety of demographic information were collected. For example, respondents reported credentials, years in clinical practice, gender, age, clinical practice sites, and states where they practiced. For all calculations, descriptive statistics were calculated for behavior analysts and trainees.

#### Workload and Compensation

An exploratory analysis was conducted to determine the average workloads for behavior analysts and trainees related to average hours worked per week, the number of assigned clients in the last year (i.e., assigned clients requiring treatment, assessment, or direct oversight and indirect oversight or supervision), and the highest number of clients they have ever been assigned. One response was removed from the calculations of the highest number of clients assigned for a year to avoid the influence of an outlier (i.e., 4000 for the year). For all calculations, descriptive statistics were calculated for behavior analysts and trainees.

Descriptive statistics were used to compare the average number of work hours behavior analysts and trainees spent on specific tasks (e.g., analyzing/graphing data, developing/writing treatment authorizations) and on categories of tasks, such as indirect tasks (i.e., job responsibilities not in the presence of a client, such as administration, training, collaboration, receiving supervision), direct tasks (i.e., direct client contact), supervision (i.e., providing supervision for others), and travel. The average number of hours spent on specific tasks within these categories were calculated by dividing the sum of the number of hours reported per task within each category by the number of respondents (e.g., the sum of the number of hours spent on analyzing/graphing data for all respondents divided by the number of respondents). The percentage of the work week spent on categories of tasks was calculated by adding the average hours for specific tasks within a category (e.g., indirect = analyzing/graphing data + developing/writing behavior plans + completing administrative duties, etc.) dividing the sum by the average total number of hours for the entire work week and multiplying by 100.

Percentages were used to describe the types of payment methods (e.g., part-time, full-time, overtime), the compensation rates (i.e., not paid, paid at full rate, paid at partial rate), and the types of tasks compensated (e.g., administration, travel, supervision) for behavior analysts and trainees. For example, the percentage of part-time pay structure was calculated by dividing the number of behavior analysts reporting that they were paid with a part-time pay structure by the total number of behavior analysts x 100.

#### Relationships Among Workload and Client Complexity

Across both behavior analysts and trainees, multiple linear regression analyses were conducted to evaluate the extent to which the number of clients within each domain of complexity (e.g., severe problem or challenging behavior, non-severe behavior, skill acquisition and levels, medical fragility, specialty skill instruction, classroom-wide intervention) could predict variables related to workload (number of reported hours worked per week, number of assigned clients for direct oversight for supervision and treatment, number of assigned clients for indirect oversight/supervision).

#### Reported Work and Personal Life Stressors Related to Workload

Since only approximately 43% of behavior analysts (*n* = 106) and 30% of trainees (*n* = 20) responded across the work and personal life stressor questions, all responses related to work and personal life stressor variables were combined into one group. Before analyzing work and personal life stressors, respondents were categorized as having a high workload (i.e., number of hours worked per week > 1 *SD* [11 hours] or more above the mean reported number of hours worked) and an average workload (i.e., number of hours worked per week ≤ 1 *SD* [11 hours] of the mean reported number of hours worked). Percentages were then reported for each category (i.e., average and high workload) for reports of work-related issues, such as considerations of changing jobs or fields, physical illness, mental stress, etc. Percentages were calculated for each item by adding the number who indicated the issue was a significant problem divided by the number who answered the question in that category (i.e., high or average workload) and multiplying by 100.

## Results

### Participant Demographics

The majority of the respondents indicated they had worked in clinical practice at some point within the last five years (behavior analysts *n* = 132, 92%; trainees *n* = 26, 84%). Responses were included for analyses for all who reported that they were behavior analysts or trainees, not dependent upon working within a specific time. The majority (> 85%) of behavior analysts had received their credentials to practice within the last ten years (0–5 years *n* = 154, 61.3%; 6–10 years *n* = 60, 23.9%). Only approximately 15% of trainees had any type of certification (e.g., BCaBA^®^).

Demographically, the respondents (i.e., those who reported being a practicing behavior analyst or trainee) represented similar demographics to the field in general (BACB, 2025), represented 33 different states, and worked at a variety of clinical practice types. Respondents were mostly female (female *n* = 275, 83.9%; male *n* = 35, 10.7%; nonbinary *n* = 4, 1.2%) and under the age of 59 (*n* = 304, 94%, *M age* = 34 years, *SD* = 8.58). The 33 different states (*n* = 165 responding to the question) included the largest group of responses from Pennsylvania (*n* = 61, 18%); Texas (*n* = 9, 6.1%), Florida (*n* = 8, 5.4%), and California (*n* = 6, 4.1%). The remaining states represented less than 4% of respondents. The most commonly reported clinical practice sites included clinics (e.g., hospital, university, private agency (*n* = 90, 27.4%), client homes (*n* = 69, 21.0%), public/private school system (*n* = 60, 18.3%), community settings (*n* = 52, 15.9%), and private ABA consulting (*n* = 44, 13.4%). Numbers related to demographic information (e.g., gender, states, and clinical practice sites) may not always have equaled the total *N* for the study, as some respondents did not answer all of the questions and some questions allowed for multiple answers by one respondent.

### Workload

#### Hours and Number of Clients

**Fig. 1:**
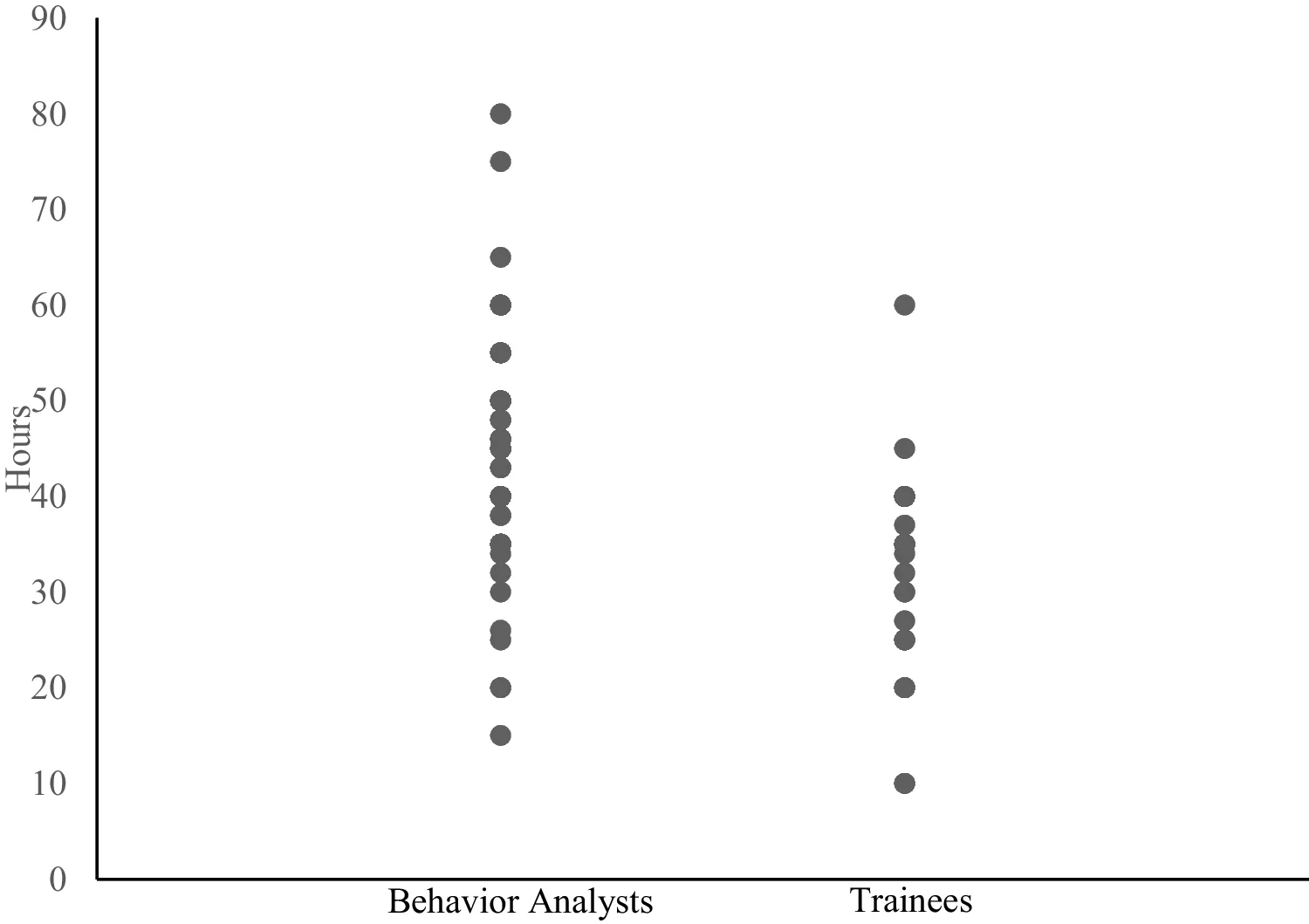
Responses for average hours worked per week

For the number of hours worked per week, behavior analysts reported on average full time or more work hour loads (*n* = 133, *M* = 45.65 hours, *SD* = 9.88, range = 15–80, *Mdn* = 45 hours [*Mdn IQR* = 10 hours]). Trainees reported slightly less than a 40-hour work week (*n* =32, *M* = 33.44 hours, *SD* = 10.31, range = 10–60, *Mdn* = 35 hours [*Mdn IQR* = 15 hours]). See Fig. 1 for a scatter plot of the average number of hours worked per week for behavior analysts and trainees.

**Fig. 2:**
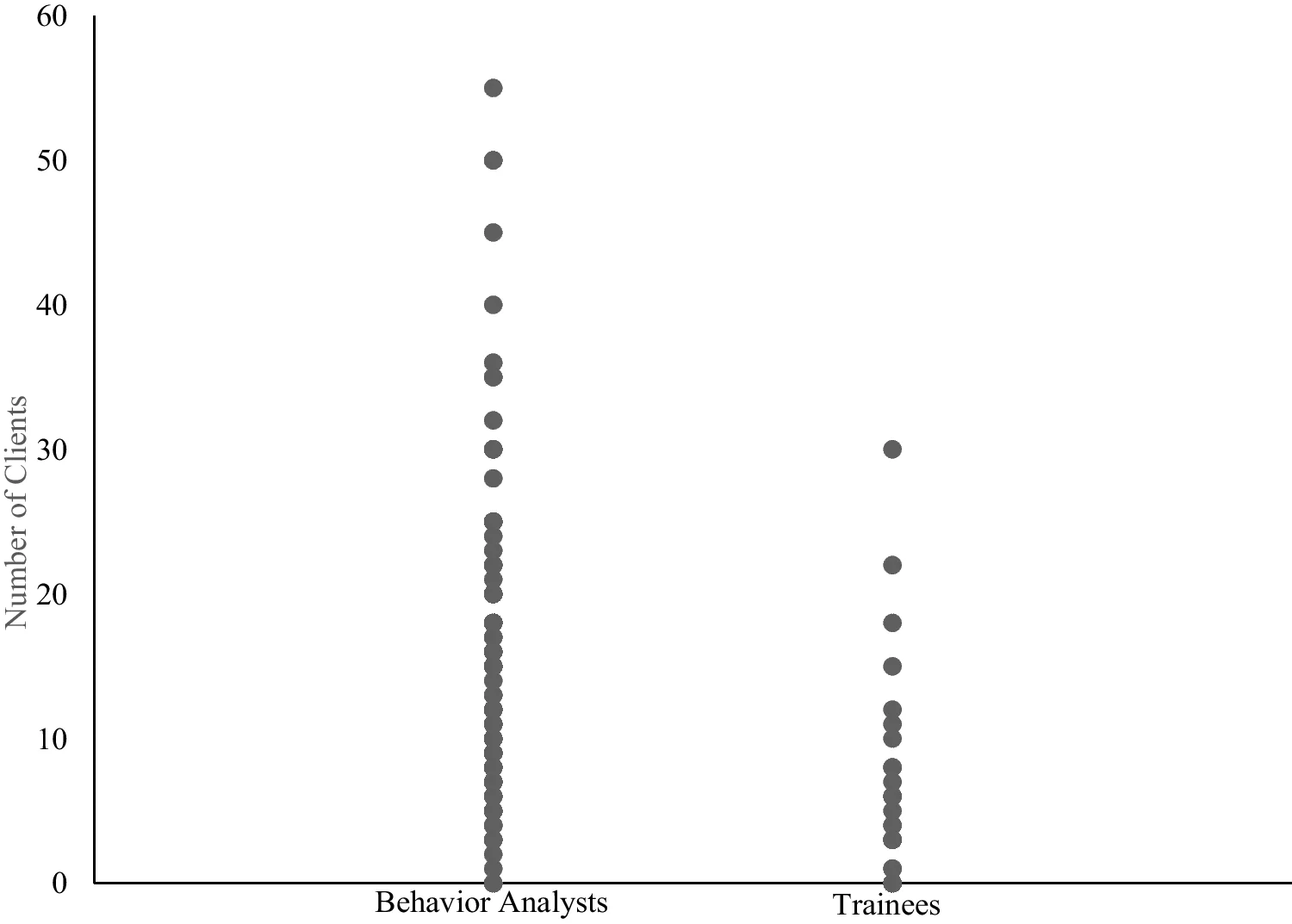
Number of clients responsible for direct oversight in last year

In the examination of the total assigned clients within the last year, behavior analysts indicated responsibility for client-based tasks, such as assessments, treatments, and direct oversite for on average 15 clients (*n* = 123, *M* = 15.11, *SD* = 10.28 range = 0 to 55, *Mdn* = 13 [*Mdn IQR* = 11]). Trainees held these responsibilities for on average approximately 6 clients (*n* =31, *M* = 6.00, *SD* = 7.22 range = 0 – 30. *Mdn* = 4 [*Mdn IQR* = 8]). Figure 2 provides a scatter plot of the average number of reported clients assigned for direct oversight within the last year for behavior analysts and trainees. Results indicated that the highest assigned number of clients for which behavior analysts had ever had responsibility for assessment, treatment, and direct oversight at one specific time ranged from 0 to 70 (*n* = 126, *M* = 15.98, *SD* =10.32, *Mdn* = 14.5 [*Mdn IQR* = 10]). The highest number of assigned clients ever for trainees ranged from 0–20 (*n* = 28, *M* = 5.21, *SD* = 5.41, *Mdn* = 3.5 [*Mdn IQR* = 4]).

In addition to having direct oversight of cases, behavior analysts reported primary responsibility for supervision or indirect oversight (e.g., consultation) for similar numbers of cases as they provided direct oversight. On average, behavior analysts reported supervising approximately16 cases over the last year (*n* = 125, *M* = 16.26, *SD* = 14.93, *Mdn* = 12 [*Mdn IQR* = 17]). Trainees reported having indirect oversight or supervision responsibilities for approximately three cases over the last year (*n* = 30, *M* = 3.30, *SD* = 10.03, *Mdn* = 0 [*Mdn IQR* = 1]).

#### Hours Spent on Specific Tasks

**Fig. 3:**
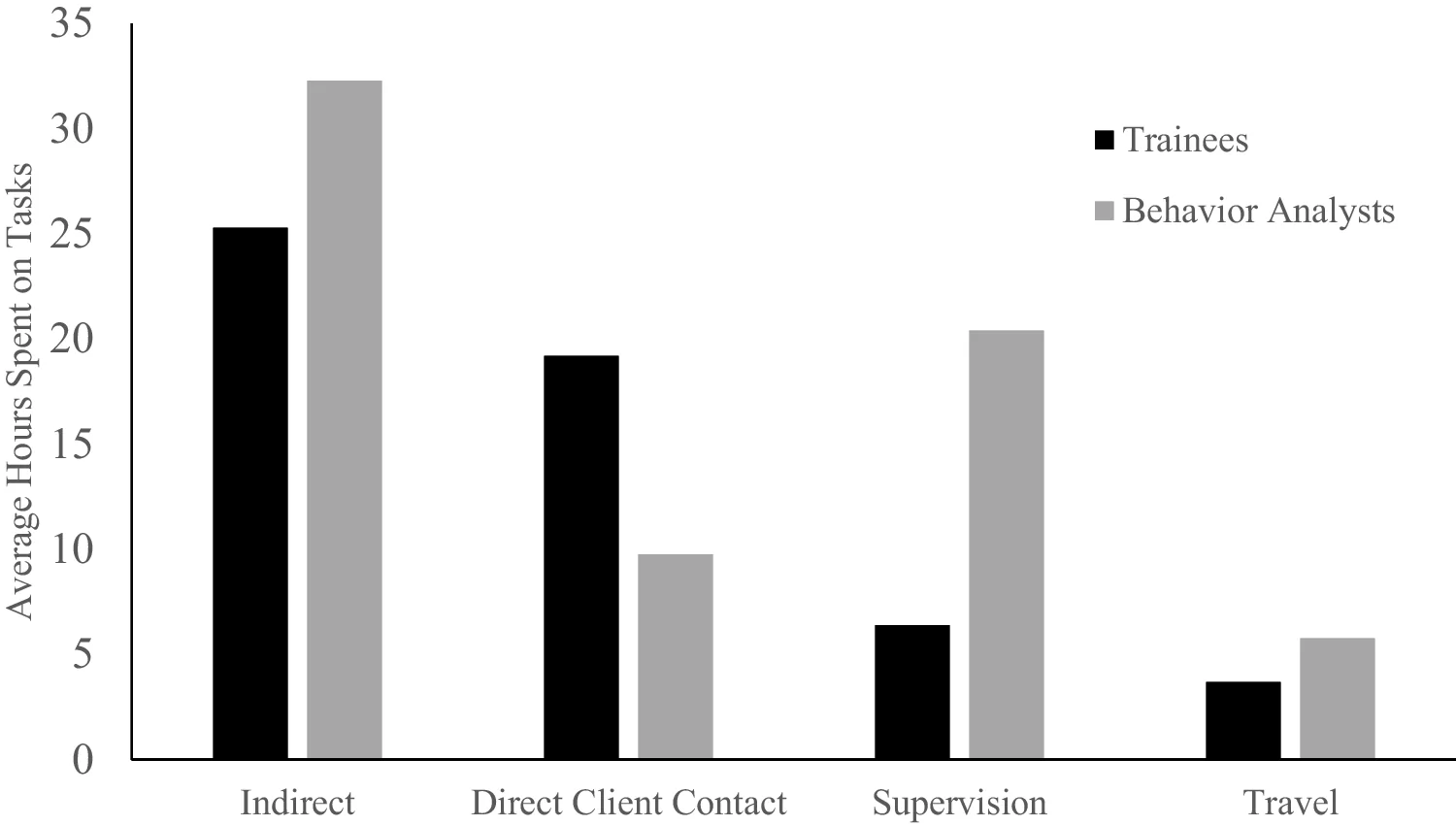
Average hours spent on tasks per week

Table 1 provides the number of respondents for each specific variable, the average hours per week, and the range for individual items within the categories for both behavior analysts and for trainees. Figure 3 represents the average number of hours spent on types of tasks within categories (i.e., indirect, direct, supervision, travel). Results indicated that behavior analysts spent the most time engaged in indirect tasks (i.e., job responsibilities not in the presence of a client) compared to direct tasks (i.e., job responsibilities in the presence of a client). Indirect tasks (i.e., paperwork and administration, personal training, and case collaboration) required the most time for behavior analysts (i.e., 46% of their work hours/week). Other activities required a significantly smaller proportion of behavior analysts’ allocated time of their hours per work week (i.e., providing supervision to staff and trainees = 29%; direct service to clients = 14%; travel = 8%). The two highest allocations of work time for trainees were indirect tasks (i.e., 46% ) and direct client care (i.e., 35%). Other activities required a significantly smaller proportion of trainees’ allocated time (i.e., providing supervision to staff and trainees = 12%; travel = 7%).

**Table 1:** Reported average hours behavior analysts and trainees spend on tasks

Item	Behavior analysts			Trainees		
	*M (SD)*	*n*	Range	*M (SD)*	*n*	Range
**Paperwork and Administration**						
Analyzing/graphing data	5.41 (5.70)	117	1–30	3.69 (6.06)	26	1–32
Developing/writing behavior plans	5.01 (4.19)	113	1–20	3.57 (2.82)	21	1–10
Developing/writing treatment authorizations	4.45 (4.19)	84	1–20	3.33 (3.32)	6	1–10
Completing administrative duties (emails, phone calls, scheduling, non-clinical paperwork, etc.)	7.42 (5.27)	118	1–25	3.29 (2.51)	24	1–10
**Direct Client Services**						
Providing direct service to clients	9.74 (8.97)	95	1–35	19.19 (12.3)	27	2–37
**Providing Supervision**						
Providing supervision to staff and RBTs^®^	16.24 (10.2)	111	1–40	6.38 (5.12)	8	2–15
Providing supervision to BCBA^®^/BCaBA^®^ trainees	4.16 (3.61)	74	1–20	0 (0)	0	0
**Personal Training**						
Receiving personal supervision	1.23 (.563)	78	.5–3	2.97 (2.29)	29	1–10
Reviewing literature on evidence-based practice	2.09 (1.49)	99	1–10	2.62 (1.83)	21	1–8
**Travel**						
Traveling	5.77 (5.18)	78	1–30	3.69 (2.44)	16	1–10
**Collaboration**						
Collaborating or consulting with professionals or mentors	3.03 (3.58)	109	.5–20	3.96 (5.00)	24	1–25
Collaborating with or training families or caregivers	3.63 (2.45)	108	1–15	2.13 (1.45)	16	1–5

#### Compensation for Workload

The results for payment for behavior analysts and trainees illustrated distinct compensation patterns for how they were paid. For behavior analysts, the primary compensation was a full-time salary with benefits (*n* = 112, 84.8%). Differing compensations rates were reported by fewer behavior analysts (full time hourly without benefits *n* = 8, 6.1%; part-time compensation salary or hourly, *n* = 8, 6.1%; contracted by hour not full time *n* = 4, 3%). Trainees reported more full-time compensation (full-time salary or hourly with benefits *n* = 20, 62.5%) as compared to part-time pay structure (salary or hourly with benefits *n* = 2, 6.2%; salary or hourly without benefits *n* = 8, 25%) or unpaid fieldwork (*n* = 2, 6.3%). For both behavior analysts and trainees, more than one-half received overtime compensation (behavior analysts *n* = 115, 87.7%; trainees *n* = 15, 62.5%).

The results for personal compensation of behavior analysts and trainees showed specific categories of tasks may not be directly compensated. See Table 2 for the number of respondents and percentage of compensation rates (i.e., not paid, paid at full rate, paid at partial rate) for individual tasks performed by behavior analysts and trainees. The majority of behavior analysts reported full compensation for direct client services (*n* = 67, 92% of respondents), administrative duties (*n* = 52, 71%), collaboration (with colleagues *n* = 50; with families *n* = 65; across all collaboration 75%), and providing supervision (supervision to staff *n* = 66; supervision to trainees *n* = 45; across all supervision 79%). However, fewer behavior analysts reported full pay for personal training (receiving supervision *n* = 39; reviewing literature *n* = 41; across all training 57%) and travel (*n* = 33, 48%). The most common individual tasks reportedly not included or only partially included within salaried job requirements were completing administrative duties (emails, phone calls, scheduling, non-clinical paperwork, etc. *n* = 32, 38.1%), receiving personal supervision (*n* = 24, 37.9%), reviewing literature on evidence-based practice (*n* = 38, 48.2%), collaborating/consulting with professionals or mentors (*n* = 28, 35.9%), and travel (*n* = 36, 52.2%). The specific tasks most likely to receive full compensation for the majority of behavior analysts were analyzing/graphing data (*n* = 63, 71.5%), developing/writing treatment authorizations (*n* = 49, 71.0%), and developing/writing behavior plans (*n* = 67, 80.7%).

**Table 2:** Reported payment behavior analysts and trainees received for tasks

Item	Behavior analysts				Trainees			
	Not paid *n* (%)	Paid at full rate *n* (%)	Paid at partial rate *n* (%)		Not paid *n* (%)	Paid at full rate *n* (%)	Paid at partial rate *n* (%)	
**Paperwork and Administration**								
Analyzing or Graphing data	15 (17.0)	63 (71.5)	10 (11.3)		8 (27.0)	13 (44.8)	8 (27.5)	
Developing or writing treatment authorizations	12 (17.3)	49 (71.0)	8 (11.5)		10 (62.5)	39 (18.7)	3 (12.9)	
Developing or writing behavior plans	8 (9.63)	67 (80.7)	8 (9.63)		12 (54.5)	4 (18.1)	6 (27.2)	
Completing administrative duties (emails, phone calls, scheduling, non-clinical paperwork, etc.)	23 (27.3)	52 (61.9)	9 (10.7)		9 (40.9)	6 (27.2)	7 (31.8)	
**Direct Client Services**								
Providing direct service to clients	2 (2.73)	67 (91.7)	4 (15.3)		4 (84.6)	22 (84.6)	N/A	
**Providing Supervision**								
Providing supervision to staff and RBTs^®^	7 (9.09)	66 (85.7)	4 (5.19)		9 (52.9)	5 (29.4)	3 (17.6)	
Providing supervision to BCBA^®^/BCaBA^®^ trainees	14 (22.2	45 (71.4)	4 (6.34)		11 (78.5)	2 (14.2)	1 (7.14)	
**Personal Training**								
Receiving personal supervision	16 (25.3)	39 (61.9)	8 (12.6)		9 (32.1)	12 (42.8)	7 (25.0)	
Reviewing literature on evidence-based practice	30 (37.9)	41 (51.8)	8 (10.1)		15 (62.5)	4 (16.6)	5 (20.8)	
**Travel**								
Traveling	23 (33.3)	33 (47.8)	13 (18.8)		16 (66.6)	2 (8.33)	6 (25.0)	
**Collaboration**								
Collaborating or consulting with professionals or mentors	21 (26.9)	50 (64.1)	7 (8.97)		11 (44.0)	9 (36.0)	5 (20.0)	
Collaborating with or training families or caregivers	3 (4.0)	65 (86.6)	7 (9.33)		8 (44.4)	8 (44.4)	2 (11.1)	

For trainees, the most common category of tasks receiving full compensation was direct client services (*n* = 22, 85%). The next highest fully compensated was for collaboration (with professionals *n* = 9; with families *n* = 8; across all collaboration 40%), personal training (receiving supervision *n* = 12; reviewing literature *n* = 4; across all personal training 22%), administrative duties (*n* = 6, 27%), and providing supervision (to staff *n* = 5; to other trainees *n* = 2; average supervision time 22%). Trainees received little compensation for travel (*n* = 2, 8%). The only specific task likely to receive full compensation for the majority of trainees was providing direct service to clients (*n* = 22, 85%). Individual tasks where trainees most likely did not receive pay or only partial compensation involved developing and writing behavior plans (*n* = 12, 81.7%), reviewing literature on evidence-based practice (*n* = 15, 83.3%), collaborating/consulting with professionals or mentors (*n* = 11, 64.0%), and travel (*n* = 16, 91.6%).

#### Relationships Among Workload and Client Complexity

Only two client complexity variables (i.e., severe behavior and medically fragile) indicated significant relationships to workload. Regression results showed a statistically significant relationship with the number of clients requiring direct oversight and supervision, *F* (8, 39) = 5.082, *p* < 0.001. The *R*^2^ was 0.51, indicating that the independent variable (i.e., complexity variables) explained 51% of the variance in the dependent variables (i.e., number of assigned clients for direct oversight). Behavior analysts who worked with and supervised clients who exhibited more severe behavior were assigned *more* clients (*b* = 1.804, *p* < .001). However, those working with and supervising medically fragile clients were assigned *fewer* clients (*b* = −1.068,* p* < .001). The correlational analysis revealed no significant correlation between the number of hours worked per week, number of assigned clients for indirect oversight, and client complexity (e.g., severe problem or challenging behavior, medical fragility).

#### Reported Work and Personal Life Stressors Related to Workload

Comparisons of the average (*n* = 120) and high workload (*n* = 28) respondents indicated some similarities and some differences. Table 3 displays the number of respondents for each issue and the percentage of significant work and personal life stressors caused by workload. For both average and high workload respondents, 50% or more of respondents reported reduced job satisfaction (*n* = 93), considering changing jobs or fields (*n* = 78), and decreased quality of work (*n* = 84). More than 75% of both groups reported mental stress (*n* = 116). One difference between the groups was the report of physical illness. For the average workload group, just over 50% reported physical illnesses (*n* = 70) related to workload. However, 75% of the high workload respondents (*n* = 21) indicated experiencing physical illness due to workload. Although respondents indicated consistently high levels of issues related to workload, less than 33% viewed workload as an ethical dilemma (average workload: *n* = 38; high workload: *n* = 7).

**Table 3:** Percent of behavior analysts and trainees reporting issues due to workload

Issues	Average Workload		High Workload	
	*n*	*%*	*n*	*%*
Considerations of changing jobs or fields	63	52.5	15	53.6
Changing of job location or employment role	59	49.2	19	67.9
Avoiding work (e.g., procrastination)	64	53.3	15	53.6
Decreased quality of your work	66	55.0	18	64.3
Personal ethical dilemma(s)	38	31.7	7	25.0
Physical illness (e.g., sleep deprivation)	70	58.3	21	75.0
Mental Stress	93	77.5	23	82.1
Reduced Job Satisfaction	75	62.5	18	64.3
Personal Issues (e.g., work–family balance)	84	70.0	23	82.1

*Note:* High workload = number of hours worked per week > 1 Standard Deviation [11 hours] or more above the 43 hour mean reported number of hours worked. Average workload = number of hours worked per week ≤ 1 Standard Deviation [11 hours] of the 43 hour mean reported number of hours worked

## Discussion

The purpose of this exploratory research was to describe (a) behavior analysts’ and trainees’ reported average workloads (e.g., work hours, numbers of client assignments, and time allocation to types of tasks) and compensation patterns, (b) relationships among workload and client complexity (e.g., behavior, goals, interventions), and (c) relationships among workload and work and personal life stressors. In general, results indicated that behavior analysts and trainees reported high workloads.

Behavior analysts reported that they worked on average more than the typical full-time 40-hour work week with some reaching more than 70 hours per week. Within these extended work hours, behavior analysts on average were assigned the high-end of the range for recommended number of assigned clients (CASP, 2020, 2024; Syed, 2024; Yingling et al., 2022). However, one-third of the respondents reported having *over* the high-end recommended number of assigned clients (i.e., 15 clients) and more than three-quarters reported having more than the middle range of recommended number of assigned clients (i.e., 7 clients). For those respondents reporting the highest number of assigned clients within this study, this indicated a client assignment rate almost 300% higher than the high-end recommendations and almost 1300% higher than the low-end recommendations (i.e., reported client numbers − recommended client hours/recommended client hours x 100). These numbers should be interpreted with caution as client number suggestions have been historical and have been substituted with case hours assuming BCaBA^®^ assistance on cases (CASP, 2020; Syed, 2024; Yingling et al., 2022). However, the number of hours reported worked per week, could suggest that case hours also exceed recommendations, especially if BCaBAs^®^ remain unassigned to cases (CASP, 2024).

Analysis of workload for trainees indicated average work hours higher than the 30-hour work week ceiling required within the BACB fieldwork requirements (BACB, 2019). The average yearly number of assigned clients for a trainee fell within the recommended range (i.e., 5–14 clients) for a *behavior analyst* (CASP, 2020; Syed, 2024; Yingling et al., 2022). Evaluating the highest report of assigned client numbers, trainees may be assigned up to 650% the lower recommended range and slightly more than 100% more than the higher recommended case numbers for *behavior analysts*. These high-end estimates remain concerning. Trainees may require fewer clients than behavior analysts because they may be extended by clients beyond or pushing their competence levels. Trainees' high-end case load estimates also may supersede their supervisors’ ability to supervise them based upon the supervisors’ workloads reported by behavior analysts. Of additional note, trainees also may be completing academic coursework while acquiring supervision hours. This increases the total number of hours engaging in professional activities, though this was not directly assessed within this survey.

Analysis of the proportion of time behavior analysts and trainees spent completing different job responsibilities indicated significant responsibilities beyond the basic client responsibilities indicated in the BCBA^®^ job description (BACB, 2024a, b) and more than those indicated in prior research and recommendations for responsibilities for time allotments (CASP, 2024; Layden et al., 2024). Using recommendations that behavior analysts could allocate 150 hours per month for supervision (CASP, 2024), behavior analysts working a 40-hour work week would be expected to provide approximately 35 hours per week to supervision (i.e., 87% of their work hours). However, additional recommendations indicated that they should spend approximately 25–30% of their time (i.e., 12 hours of a 40-hour work week) completing activities not involving direct client care or supervision (CASP, 2024). Although these recommenders provided a caveat that other issues should contribute to allocation of time (e.g., experience, travel, complexity of cases), the current recommendations provided no specific guidelines for determining time allocation. Thus, employers may attempt to rely upon the specifically provided hour recommendations which could result in a behavior analyst’s work week significantly beyond 40 hours per week (e.g., 35 hours/week recommended direct oversight + 12 hours of recommended indirect hours + 5 or more hours of reported travel = 52-hour work week.

In addition to recommendations providing time to task allocations beyond a typical work week, findings from this study indicated a significant discrepancy from the recommended percentage of time estimate allotments (CASP, 2024) to the time that behavior analysts actually reported spending on specific tasks. In contrast to the estimates of 25–30% of their time (i.e., 12 hours of a 40-hour work week) recommendation estimate allocation for indirect activities, behavior analysts reported approximately 46% of their time allocated to indirect activities (e.g., data analysis, treatment plan development, treatment-based research review, client-specific collaboration). Additionally, in contrast to the 87% of time estimate allocations recommended for direct oversight and supervision of cases, behavior analysts reported only 29% of their time providing supervision.

Research and recommendations previously have indicated that high workload hours have impacted service and supervision quality, health, and turnover/attrition rates, especially for complex clients (Bottini et al., 2020; Brown, 2021; CASP, 2020, 2024; Dounavi et al., 2019; Jimenez-Gomez et al., 2021; Kazemi et al., 2022; Kranak, 2022; Plantiveau et al., 2018; Slowiak & DeLongchamp, 2022; Slowiak & Jay, 2023; Syed, 2024). The results of this study indicated that client complexity involving severe behavior inversely impacted assigned numbers of clients and supervision requirements (i.e., more clients with severe behavior equaled more total assigned clients for direct oversight) and that medical fragility of clients resulted in fewer assigned numbers of clients for direct oversight. This partially contradicted recommendations that complex clients should result in fewer assigned clients and supervision requirements (CASP, 2020, 2024).

With the higher than recommended assigned number of clients and work hours and significant indirect job responsibilities suggested in this study, those working in the behavior analysis field may be at high risk for work and personal life stressors (e.g., physical illness and mental stress). This study supported previous research suggesting relationships among these factors (Bottini et al., 2020; Brown, 2021; Dounavi et al., 2019; Jimenez-Gomez et al., 2021; Kazemi et al., 2022; Kranak, 2022; Plantiveau et al., 2018; Slowiak & DeLongchamp, 2022; Slowiak & Jay, 2023; Syed, 2024). More than one-half of respondents with an average workload indicated physical illnesses related to their job, while three-quarters of respondents with high workloads reported this same concern. Although no differences were found between average and high workloads for mental stress or personal life issues, job attrition, or perceived job quality, more than one-half to three-quarters of respondents indicated significant impacts in these areas related to workload.

The relationships among assigned clients, work hours, tasks, compensation, and personal issues has only been partially investigated (Kazemi et al., 2015; Novack & Dixon, 2019) and has indicated that for behavior analysts and trainees, adequate compensation may increase job satisfaction and reduce personal life issues. In this study, some of the behavior analysts reported receiving only partial compensation for every one of their job responsibility categories. The highest fully compensated area of responsibility with more than three-quarters of respondents receiving full compensation included direct client services, providing supervision, and collaboration with other professionals. Between one-third to one-half reported receiving only partial or no compensation for completing administrative duties required by a behavior analyst (e.g., analyzing, graphing, and developing and writing treatment plans). Around one-half received only partial or no compensation for travel and maintaining professional competence (i.e., personal training). For trainees completing fieldwork, this study indicated that the majority of their compensation came from direct client services and collaboration with professionals. They received little compensation for any of their other reported job responsibilities.

Although this exploratory study indicated reasons for possible concerns with behavior analysts’ and trainees’ workloads, these reported results must be interpreted with caution. The exploratory nature of this study can only provide preliminary insights and must be interpreted within the limitations of the study. As an exploratory study, a calculation of the number of required participants was not conducted. The respondents in this study represented only a small sample size of overall practicing behavior analysts and trainees with unequal proportions of behavior analysts and trainees across geographical locations. However, group and questionnaire exploratory designs typically require inferences from the sample reflecting the characteristics of the target population to allow for generalization. This study represented the overall target population of behavior analysts and trainees with the majority being female, the average age in the mid-to upper-thirties, and work sites being in education and clinical practice (BACB, 2025). Although using a representative sample of the overall respondent pool provided an acceptable methodology, this sample may have resulted in a general limitation of generalizability of results to the majority of practicing behavior analysts and trainees.

The current study also failed to collect some demographic information that could impact interpretation of results. For example, the study did not evaluate race/ethnicity of the respondents. It also did not determine if behavior analysts had a BCaBA^®^ assigned to any of their cases. A BCaBA^®^’s assistance on cases could have minorly influenced the interpretation of work overload reports because an addition of a BCaBA^®^ could increase recommended case assignments to a maximum of 24 rather than 15 for specific treatment (CASP, 2020). However, the respondents’ reports of the time spent on tasks suggested the absence of BCaBAs^®^ on their cases. Despite the presence or absence of assistance on cases, workload and recommendations for more comprehensive behavior analytic treatment also remained within the ranges used in the extrapolations above.

The current study also may have provided only a glimpse of behavior analysts’ workloads, compensation, and impacts at a single point in time. The study attempted to explore caseloads across the last year to provide a more representative report of hours worked. This decision was made to attempt to reflect an overall picture of behavior analysts’ and trainees’ work hours and tasks, which would possibly be less reflective of a temporal schedule (e.g., recent, temporary reduction or increase in case load or responsibilities). Although the decision to explore work requirements over the last year was made to provide a more global representation, respondents were not provided with dates or instructions on how to determine the year. Thus, responses could have been reflective of environmental occurrences differently across respondents.

In addition to the temporal responsivity of the responses, the types of questions, self-report method of the questionnaire, and the online format also could have introduced some difficulty with question interpretation, possible bias (e.g., memory), or bot responders. For example, for exploring tasks not requiring direct client contact, the questionnaire described these activities as non-face-to-face time. The use of this description may have caused ambiguity and thus altered responses. Additionally, data was not assessed or cleansed for possible bot responders. All limitations within this study, such as the limited number of respondents in this study, the inability to evaluate some demographics related to the representativeness of the respondent sample, and the pinpointed self-report time analysis may have resulted in a non-comprehensive representation of the diversity of the behavior analyst profession.

Despite these limitations, this study provides a possibly concerning pattern with ethical implications that indicate the need for further research. If replication indicates support of the patterns reported in this study, behavior analysts’ and trainees’ workloads may have significant implications related to the Ethical Code for Behavior Analysts’ (BACB, 2020) Section 1 (Responsibility as a Professional), Section 2 (Responsibility in Practice), Section 3 (Responsibility to Clients and Stakeholders), and Section 4 (Responsibility to Supervisees and Trainees). Reports of significantly high numbers of assigned clients and additional workload responsibilities combined with reports that these factors influenced work quality, indicated significant issues, especially because respondents appeared conditioned to not connect workload impacts as a potential ethical dilemma.

Individuals, organizations, and the field must continue to assess and evaluate workload conditions, conduct frequent discussions, and continue to emphasize the interaction of workload and ethical issues. Within these discussions, interventions could occur involving prevention, assessment, and intervention for work and personal life stressors at the individual behavior analyst/trainee, organizational, and field levels. These interventions could provide strategies for promoting balance between work and personal life. Key approaches include training in time management, regularly assessing workload and competencies, adjusting responsibilities to align with individual skill levels, and fostering supportive work environments. At the individual, organizational, and industry levels, efforts should focus on equitable workloads, ongoing professional development, and aligning job demands with compensation. See Table 4 for references and suggestions for prevention, assessment, and intervention actions at each level. If each of these levels refrain from assessing and adjusting workloads exceeding recommendations and the limits of what individual behavior analysts and trainees can manage, the effectiveness of our interventions will suffer, clients will suffer, our professionals will suffer, and ABA as a field may ultimately suffer.

**Table 4:** Reducing ethical issues related to caseload

	Suggestions
**Prevention of Work and Personal Life Impacts & Possible Ethical Implications**	
	Include teaching of strategies to prevent work and personal life impacts during academic training and fieldwork^2,3^ (Dounavi et al., 2019; Kranak, 2022)
	Require assessment, instruction, supervision, and implementation of time management for work and personal care^1,2,3^ (Dounavi et al., 2019; Fiebig et al., 2020; Slowiak & DeLongchamp, 2022)
	Consistently assess workhours and responsibilities^1,2,3^ (Dounavi et al., 2019)
	Adjust workloads according to responsibilities and work/life requirements^1,2,3^ (Dounavi et al., 2019)
	Establish guidelines, equity, and compensation for workload, “values,” and actual job responsibilities^2,3^ (Bottini et al., 2020)
	Assess and match job responsibilities and difficulty to individual’s competencies^1,2^ (Otto et al., 2021; Slowiak & DeLongchamp, 2022)
	Obtain continued professional education to maintain and expand competence^1,2,3^ (Bottini et al., 2020; Novack & Dixon, 2019; Slowiak & DeLongchamp, 2022)
	Develop coworker support systems^1,2^ (Dounavi et al., 2019; Otto et al., 2021, Plantiveau et al., 2018; Slowiak & DeLongchamp, 2022)
	Increase frequency and maintain supervisor/mentor support^1,2^ (Dounavi et al., 2019; Novack & Dixon, 2019; Otto et al., 2021: Plantiveau et al., 2018)
	Allow employees self-scheduling and autonomy in job completion^2^ (Otto et al., 2021)
	Assess motivating operations, “values”, and discriminative stimuli for work and self-care^1^ (Bottini et al., 2020; Slowiak & DeLongchamp, 2022)
	Develop personal life support systems^1^ (Otto et al., 2021)
	Reduce unnecessary or non-reinforcing work and after work obligations^1,2^ (Kranak, 2022; Otto et al., 2021; Slowiak & DeLongchamp, 2022)
	Conduct preference assessments related to workload^1,2^ (Bottini et al., 2020)
	Improve physical and mental health^1^ (Otto et al., 2021)
**Assessment Related to Work and Personal Life Impacts**	
	Consistently assess relationship of workhours to responsibilities^1,2^ (Bottini et al., 2024)
	Assess resources delegated to work/life responsibilities^1^ (Otto et al., 2021)
	Assess case and task list difficulty level^1,2^ (Bottini et al., 2024)
	Asses clinical competency as it relates to each case (BACB, 2020; Brodhead et al., 2018)
	Develop and assess indicators of work and personal life impacts, such as making mistakes, missing deadlines, etc.^1,2^ (Bottini et al., 2024; Fiebig et al, 2020)
**Intervention for Work and Personal Life Impacts**	
	Evaluate and adjust individual’s responsibilities related to prevention areas above^1,2^ (Bottini et al., 2024; Otto et al., 2021)
	Adjust work hours and/or job demands^1,2^ (Brashear, 2022; Otto et al., 2021; Slowiak & DeLongchamp, 2022)
	Alter or block out meeting times to preferred times of day^1,2^ (Brashear, 2022; Slowiak & DeLongchamp, 2022)
	Vary clients based upon behavior and complexity of cases^1,2^ (Brashear, 2022; Slowiak & DeLongchamp, 2022)
	Reduce non-reinforcing non-client activities^1,2^ (Brashear, 2022; Slowiak & DeLongchamp, 2022)
	Increase equitable relationship between workload and reinforcement (e.g., compensation and recognition)^2^ (Bottini et al., 2020; Dounavi et al., 2019; Kranak, 2022)

*Notes*: Superscripts indicate activity recommendations at the Individual Behavior Analyst or Trainee Level^1^, Organizational Level^2^, and Industry/Field Level^3^

## Supplementary Information

Below is the link to the electronic supplementary material.

Supplementary file1 (DOCX 33 KB)

## Data Availability

Data will be made available upon reasonable request.
