# Supplementary material for: Behavior Analyst & Trainee Workloads: Baseline Reports, Ethical Implications, and Practical Solutions
Source: Behav Anal Pract. 2025 Dec 10;19(3):993–1005. doi: 10.1007/s40617-025-01113-5 (PMC13638835; doi:10.1007/s40617-025-01113-5)
Supplement: Supplementary file 1 [file 40617_2025_1113_MOESM1_ESM.docx]

Appendix

Questionnaire

Q1 Which of the following best describes your credentials for practicing behavior analysis (select all that apply)?

BCBA^®^

BCBA-D^®^

Licensed Psychologist

State Licensed Behavior Analyst (or equivalent)

BCBA^®^ Trainee (e.g., ABA student, doing Fieldwork)

None of the above

Q2 How long ago did you receive your BACB certification or credentials to practice?

Not yet certified, currently supervised

<2 years

2-5 years

6-10 years

10+ years

Q3 How old are you?

Q4 What is your gender identification?

Male

Female

Nonbinary

Prefer not to say

Other __________________________________________________

Q5 Which of the following best describes your current clinical practice environment. Please select all that apply.

Public school system

Private school system

Specialized school for children with ASD or developmental disabilities

Special education unit or classroom

Non-special education classrooms

Private ABA consulting (individual or agency)

Group homes

Client homes

Community settings

Residential Treatment Facilities

Clinics (e.g., Hospital, University, Private Agency)

Other __________________________________________________

Q6 In what state do you primarily practice?

Select One:

▼ Alabama ... Wyoming

Q7 Have you worked in ABA clinical practice in the last five years?

Yes

No

Q8 How do you get paid for your clinical work?

Full Time Salary (with benefits - for example, paid time off)

Full Time Hourly (with benefits - for example, paid time off)

Part Time Salary (with benefits - for example, paid time off)

Part Time Salary (without benefits)

Part Time Hourly (with benefits - for example, paid time off)

Part Time Hourly (without benefits)

Contracted (i.e., paid by hour but no benefits)

Unpaid (fieldwork)

Q9 Over the last year, how many hours per week do you work?

________________________________________________________________

Q10 Do you get paid overtime/bonus for working more than 40 hours?

Q11 Across all your clients, how many hours do you spend per week engaged in the following non-face-to-face

activities?

|  | **Average Number of Hours Per Week** | **Source of Personal Reimbursement** | | | **Check if activity counts as billable hour?** |
| --- | --- | --- | --- | --- | --- |
|  |  | **Not paid for activity** | **Paid at Full Rate** | **Paid at Reduced Rate** |  |
| Analyzing/graphing data |  |  |  |  |  |
| Developing/writing behavior treatment plans |  |  |  |  |  |
| Developing/writing treatment authorizations |  |  |  |  |  |
| Providing direct service to clients |  |  |  |  |  |
| Providing supervision to staff and RBT^®^s |  |  |  |  |  |
| Providing supervision to BCBA^®^/BCaBA^®^ trainees |  |  |  |  |  |
| Receiving personal supervision |  |  |  |  |  |
| Traveling |  |  |  |  |  |
| Collaborating/consulting with professionals or mentors |  |  |  |  |  |
| Collaborating with or training families or caregivers |  |  |  |  |  |
| Completing administrative duties (emails, phone calls, scheduling, non-clinical paperwork, etc.) |  |  |  |  |  |
| Reviewing literature on evidence-based practice |  |  |  |  |  |
| Other |  |  |  |  |  |

Q12 How many clients within the past year have you been primarily responsible for assessment, treatment, or direct oversite?

________________________________________________________________

Q13 How many clients within the last year have you been primarily responsible for supervision or indirect oversight (e.g., consultation or supervision of other's direct work)?

________________________________________________________________

Q14 What was the highest number of clients in your caseload (i.e., you ever were assigned to directly construct or conduct treatment with) at one specific time?

________________________________________________________________

Q15 How many cases in your caseload over the past year involve any of the following? (If more than 250, move the slider to the maximum)

|  | More than 250 |
| --- | --- |

| Severe problem behavior (aggression/sib) |  |
| --- | --- |
| Non-severe behavior (elopement, non-significant property destruction, screaming) |  |
| Medically Fragile |  |
| Nonverbal |  |
| Skill acquisition goals |  |
| Advanced communication or problem solving goals |  |
| Specialty skill instruction (feeding, toileting, etc.) |  |
| Classroom-wide interventions |  |

Q16 Has your workload resulted in any of the following being a significant issue for you (select all that apply)?

Consideration of changing jobs or fields

Changing of job location or employment role

Avoiding work (e.g., refusal to taking new responsibilities, procrastination, etc. )

Decreased quality of your work (e.g., mistakes, missing deadlines, poor performance)

Reduced job satisfaction

Personal ethical dilemma(s)

Physical illness (e.g., sleep deprivation, headaches, fatigue, exhaustion, etc.)

Mental Stress

Personal Issues (e.g., work family balance, lack of self-care, etc.)

Other __________________________________________________

We appreciate the time you spent on this survey. Is there anything you would like to tell us related to behavior analysts' workload ?

________________________________________________________________
